# An interesting case of chronic myeloid leukemia (CML) with T315I mutation raising suspicion of de novo AML, a diagnostic conundrum

**DOI:** 10.1002/ccr3.5908

**Published:** 2023-05-23

**Authors:** Phool Iqbal, Aamir Shahzad, Zubair Shahid, Firdous Ghori, Halima Elomri, Dina Soliman

**Affiliations:** ^1^ 36977 Department of Internal Medicine and Medicine Critical Care Department Hamad Medical Corporation Doha Qatar; ^2^ Department of Internal Medicine Readings Hospital Tower Health Reading Pennsylvania USA; ^3^ 36977 Department of Internal Medicine and Cardiology Hamad Medical Corporation Doha Qatar; ^4^ Medical Oncology‐Hematology Department National Centre for Cancer Care and Research (NCCCR) Hamad Medical Corporation (HMC) Doha Qatar

**Keywords:** *BCR*‐*ABL1*+ AML, chronic myeloid leukemia, de Novo AML, Philadelphia chromosome, T315I

## Abstract

Chronic myeloid leukemia (CML) is a myeloproliferative disorder due to translocation between chromosomes (9, 22), known as the “Philadelphia chromosome.” In 2016, the World health organization (WHO) introduced a new clinical entity of de novo acute myeloid leukemia (AML). Both diseases share some commonalities, therefore, create a challenge to diagnose.

## Abstract 2

The differentiation of CML‐BP from de novo AML is crucial in the management of the patient. Therefore, a cautious diagnostic approach involving hematologists and hemato‐histopathologist is required where there is a diagnostic dilemma.

## 1 INTRODUCTION

CML is a myeloproliferative neoplasm characterized by a translocation between chromosomes (9, 22), resulting in a BCR‐ABL1 fusion gene with tyrosine kinase activity. The CML‐BP can transform into acute lymphoblastic leukemia (ALL) as well as acute myeloblastic leukemia.^1^ In 2016, WHO has included AML with BCR/ABL1+ gene as a separate provisional entity in its latest classification of myeloid neoplasms.^1^, ^2^ We report a case of CML‐BP in a middle‐aged gentleman that posed a challenge to diagnose and differentiate it from de novo AML due to lack of a definite clinical criteria in such clinical scenarios.^2^

## 2 CASE PRESENTATION

A 33‐year‐old healthy, non‐smoker Bangladeshi gentleman presented with fatigue, subjective fever, bruises, and gum bleeding from 7 days without any significant weight loss or swellings on the body. He had no significant past medical history or any co‐morbid illness.

Physical examination was remarkable for conjunctival pallor and few scattered patches of ecchymosis all over the body, with the largest measuring 3 × 2 cm over the abdomen. There were petechiae on the soft palate around the tongue and a palpable spleen below the left costal margin (splenomegaly). There was no generalized lymphadenopathy. The rest of the systemic examination was normal. Initial blood investigations are shown in Table 1.

**TABLE 1:** Initial complete blood count

Lab Parameters	Results	Normal Range	Interpretation
Hemoglobin	10.7 g/dl	13–17 g/dl	Low
Platelets	79 × 10^3^/µl	150–400 × 10^3^/Ul	Low
WBCs	46.6 × 10^3^/µl	4 × 10^3^/Ul–10 × 10^3^/Ul	High

### 2.1 Peripheral smear findings

**FIGURE 1:**
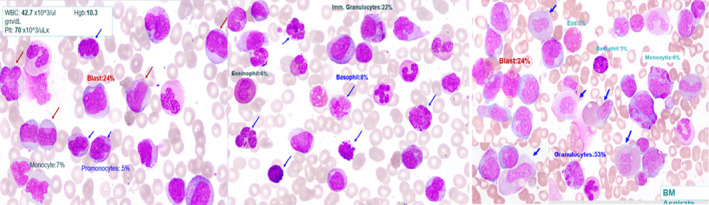
Peripheral smear showing circulating blasts, basophils, eosinophils, and dwarf megakaryocytes along with significant dysgranulopoiesis

There was mild normocytic normochromic anemia with few polychromatophilic cells, few tear‐drop cells and rare circulating nucleated red blood cells. There was leukocytosis with marked shift to left, many circulating blasts (25%), basophilia (8%), and eosinophilia. Monocytosis including abnormal monocytes and promonocytes (5%) was also seen. There was significant dysgranulopoiesis in the form of abnormal nuclear segmentation, mostly hyposegmentation, and abnormal granulation in the form of hypogranulation/ hypergranulation with an abnormal localization of cytoplasmic granulation. Platelets were moderately reduced. 300‐cell differential showed blasts: 24%, Promyelocyte: 7%, Myelocyte: 6%, Band: 9%, Segmented Neut: 21%, Lymphocytes: 6%, Monocyte: 7%, Promonocytes: 5%, Eosinophil: 6%, Basophil: 8% as shown in Figure 1.

### 2.2 Bone marrow (BM) aspirate smear findings

BM aspirate was hypercellular with multiple hypercellular particles. There are few megakaryocytes seen, including small hypolobulated forms. Erythropoiesis appeared moderately suppressed with normoblastic maturation and some megaloblastoid changes. Granulopoiesis was active with many blasts (24%), many granulocytes in different stages of maturation and increase basophils (3%). There was significant dysgranulopoiesis in the form of abnormal nuclear segmentation, mostly hyposegmentation and abnormal granulation in the form of hypogranulation/hypergranulation, with an abnormal localization of cytoplasmic granulation. 500‐cell differential count showed erythroid precursors: 8%, Blast: 24%, Granulocytes: 53% (Promyelo: 13%, Myelo: 14%, Metamyelocyte: 1%, Band: 12%, Segmented: 12%), Basophil: 3%, Eosinophil: 7%, Monocyte: 5%, Promonocytes: 1%.

### 2.3 Immunohistochemistry

CD34: Few scattered positive cells.

CD117: Highlighted some positive cells with some clusters.

CD68: Many positive cells.

CD3: Few scattered positive cells.

CD20: Some scattered positive cells.

PAX‐5: Few scattered positive cells.

Glycophorin: Highlighted some erythroid precursors; appear suppressed.

MPO: Highlighted marked increase in granulocytic precursors.

vWF& CD61: Highlighted some megakaryocytes including some small hypolobulated forms.

### 2.4 Flow cytometry

Flow cytometry on bone marrow aspirate shows an abnormal population of immature cells comprising ~29% expressing CD45 (moderate), CD33 and CD4 (dim). The majority of these immature cells express HLA‐DR, CD64, CD13, CD15, CD11c and CD36. There is partial expression of CD11b and CD7 (aberrant). The majority of these immature cells are negative for CD34, CD117 and CD14. A small subpopulation (approximately 4%) expresses dim CD5 (aberrant). There is a small subpopulation (approximately 4%) expressing CD34 and CD117.

There is no significant expression of CD19, CD20, CD10, surface CD3, CD2, CD9, CD41, CD61, CD56, cytoplasmic CD79a, cytoplasmic CD3, cytoplasmic MPO, Glycophorin A, or TdT.

The overall findings were of blast population with monocytic immunophenotype. FISH study revealed BCR/ABL1 fusion gene in 95% of the cells.

### 2.5 Karyotype analysis

46,XY,t(9;22;17)(q34;q11;q23)[2]/46,idem,t(3;21)(q26.2;q22)[28]/47,idem, t(3;21)(q26.2;q22),+8[25].

**FIGURE 2:**
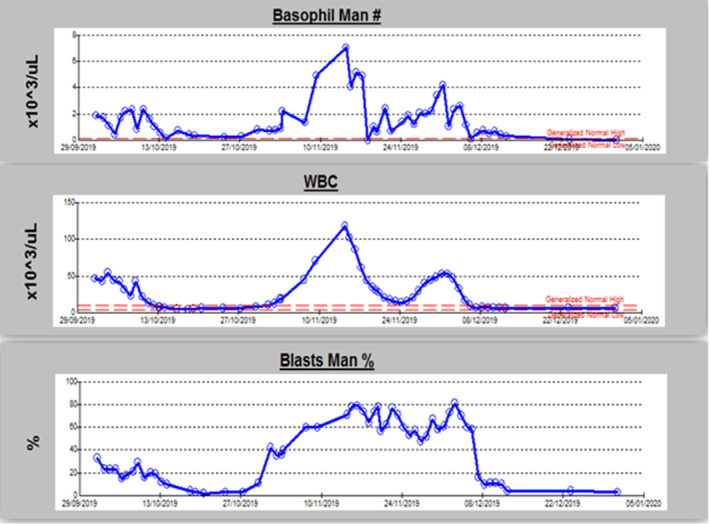
Response of the patient after ponatinib and trend in basophils, WBCs, and blast cells

Overall findings were consistent with Acute Myeloid Leukemia with monocytic differentiation with features favoring evolvement on top of CML (blast phase), but another differential diagnosis of de novo AML with BCR/ABL1 could not be excluded entirely during diagnostic workup. It was suggested initially to commence the patient on chemotherapy as per de novo AML protocol. However, after a thorough discussion with hemato‐histopathologists and senior hematologists in a multidisciplinary team (MDT) meeting, he was commenced earlier on TKI (Tyrosine kinase inhibitor therapy) as the majority of cells were suggestive of CML with BCR ABL1 fusion gene. Cytoreduction therapy with hydroxyurea and Dasatnib was started. He had a suboptimal response with an increase in blast cells after 3 weeks of therapy. It raises a concern about his diagnosis and reconsideration of de novo AML. Therefore, another MDT was held to review the patient clinical presentation and diagnostic workup. It concluded in favor of CML with evolution into acute myeloblastic phase with monocytic differentiation. As he was resistant to TKI therapy, mutation analysis for the T315I gene was sent to rationalize blast cells’ increase after Dasatinib therapy. Mutational analysis for the T315I gene turned out to be positive. Therefore, he was started on Ponatinib, a recommended TKI therapy for CML patients with a positive T315I gene. The drug was made available on special arrangements by a local hospital charity's help due to its unavailability in the country. There was a good response with a decrease in WBCs, basophil cells, and blast cells in one week, as shown in Figure 2, respectively. Further donor search was also initiated for hematopoietic stem cell transplant (HSCT), and he remained stable throughout his hospital course.

## 3 DISCUSSION

CML is a form of myeloproliferative neoplasm (MPN) characterized by a balanced chromosomal translocation t(9; 22) (q34; q11.2), also known as the Philadelphia chromosome.^1^ The resultant gene, BCR/ABL1 (breakpoint cluster region/Abelson gene), has tyrosine kinase activity that leads to abnormal growth of the cells.^2^, ^3^ CML accounts for about 15% of newly diagnosed cases of leukemia in adults.^3^

AML with BCR/ABL1+ had been included as a separate provisional entity in 2016 by WHO classification of myeloid neoplasms.^1^ AML with BCR/ABL1+ is considered to carry a worse prognosis, and hence its management approach is different from CML‐BP.^4^ There are overlapping clinical features between *BCR*‐*ABL1*+ AML and myeloid CML blast crisis; moreover, there are no definite clinical criteria established yet to distinguish among these entities.^4^, ^5^ The involvement of molecular markers such as IKZF1, CDKN2A, and antigen receptor gene deletions in IGH or TRG2 can distinguish between de novo BCR‐ABL1+ AML from myeloid blast crisis of CML.^1^, ^2^ Certain other reported clinical features in the literature can also guide in this diagnostic dilemma, as mentioned in the Table 2; however, they may not be seen in every case.^5^, ^6^

**TABLE 2:** Differentiating points described in the literature to guide between the blast crisis of CML and Denovo AML with the BCR‐ABL1 fusion gene

Blast phase of CML (CML blast crisis)	Acute myeloid leukemia with BCR‐ABL1 (provisional entity)
Splenomegaly is common^5^, ^9^	Splenomegaly less frequent^5^, ^9^
Prominent peripheral blood basophilia (usually >2% basophils)^5^, ^6^, ^8^, ^9^	Low peripheral blood basophilia (usually <2% basophils)^5^, ^6^, ^8^, ^9^
Hypercellularity of the bone marrow (BM) 95– 100%^9^	BM cellularity is reported to be less than that typically seen in blast transformation of CM^9^
Micromegakaryocytes (dwarf megakaryocytes) is a feature^9^	Dwarf megakaryocytes are less common^9^
Cytogenetic abnormalities like chromosome 7 monosomy, chromosome 16 inversions, and chromosome 10 deletions are absent^8^	Cytogenetic abnormalities like chromosome 7 monosomy, chromosome 16 inversions, and chromosome 10 deletions are present^8^
Molecular features like NPM1 mutation and p190 are less seen in CML‐BP^6^, ^8^	Molecular features like NPM1 mutation and p190 are prevalent in de novo AML^6^, ^8^

Our patient presented with clinical features of splenomegaly, peripheral circulating basophils more than 2% with blast cells, and hypercellular bone marrow supporting CML‐BP diagnosis.^6^ He had mixed cellular phenotypic variation of CML‐BP and AML with monocytic differentiation on bone marrow examination, which created another differential diagnosis of de novo AML on the table. Later after discussing the case in an MDT of hemato‐histopathologist and reviewing the patient's clinical file supplemented with cytogenetics and molecular analysis, he was labeled as a case of CML‐BP and treated accordingly with TKI therapy, that is, dasatinib but did not respond adequately. Later T315I mutation analysis came positive, and he received the recommended treatment with ponatinib therapy with optimal response.^3^ His genetic analysis did not show p190‐type or p210‐type BCR/ABL1 and NPM1 mutation although this feature is more prevalent in De Novo AML as mentioned in Table 2 but not seen in our patient and thus favoring the picture of CML‐BP more and clearing further ambiguity of diagnosing such a clinical presentation. Furthermore, Gupta et al. described the importance of differentiating CML‐BP from De Novo AML as it has treatment and prognosis implications.^7^ Overall, such a mixed presentation of CML‐BP raising suspicion of de Novo AML is a diagnostic challenge and needs multi‐disciplinary teams onboard as we managed to do in our case.

The reason to differentiate de novo AML is based upon its difference in genetic and molecular nature that poses high risk other than BCR/ABL1+ gene only, treatment modality, and response from CML.^1^, ^2^, ^3^, ^4^ Studies have also revealed that de novo AML with BCR/ABL1 has more prevalence of fusion protein 190 and NPM1 mutation in contrast to Ph+CML and also possess different treatment and prognostic value than CML with BCR/ABL1 in blast phase.^8^ After a thorough literature search, we managed to execute a table to guide the CML‐BP and de novo gene AML,Table 2.^5^, ^6^, ^8^, ^9^

In the literature, it has been mentioned that it is quite rare and challenging to diagnose CML‐BP with monocytic differentiation and therefore karyotyping and/or molecular studies to confirm the diagnosis for further TKIs targeted therapy.^10^ Although the myeloid blast phase is quite common, the monocytic blast phase of CML associated with T315I is the first case reported in our National Center for Cancer care and research institute (NCCCR) in Qatar.

## 4 CONCLUSION

We aim to highlight a diagnostic approach toward a new clinical entity of de Novo AML with BCR/ABL1 fusion gene described by WHO in 2016 from CML with BCR/ABL1 gene, which can be challenging in some clinical scenarios. And we also emphasize the need for T315I mutation analysis study in TKI resistant cases of CML to deliver optimum patient care.

## AUTHOR CONTRIBUTIONS

Phool Iqbal: Case identification and management, manuscript writing, editing and literature review. Aamir Shahzad: manuscript editing and writing with literature review. Zubair Shahid: manuscript editing and writing. Firdous Ghori: Literature review, manuscript editing and writing. Dina Soliman: Literature review, manuscript editing and supervision. Halima El Omri: Literature review, manuscript editing and supervision.

## CONFLICT OF INTEREST

The authors certify that they have no conflict of interest and no affiliations with or involvement in any organization or entity with any financial or non‐financial interest in the subject matter or materials discussed in this manuscript.

## APPROVAL FROM THE INSTITUTIONAL RESEARCH BODY

The manuscript completed the review process by the medical research council of Hamad Medical Corporation, using their online platform "www.abhath.hamad.qa."

## ETHICAL APPROVAL

The patients have given verbal consent to publish this case. The study is conducted ethically in accordance with the World Medical Association Declaration of Helsinki.

## CONSENT

Written informed consent was obtained from the patient to publish this report in accordance with the journal's patient consent policy.

## Data Availability

Authors confirm that all relevant data or information are included in the article and is available via open access platform of this journal.
